# Free-standing microscale photonic lantern spatial mode (De-)multiplexer fabricated using 3D nanoprinting

**DOI:** 10.1038/s41377-024-01466-6

**Published:** 2024-06-03

**Authors:** Yoav Dana, Yehudit Garcia, Aleksei Kukin, Lauren Dallachiesa, Sterenn Guerrier, Nicolas K. Fontaine, Dan M. Marom

**Affiliations:** 1 Institute of Applied Physics, Hebrew University of Jerusalem, Jerusalem, Israel; 2 Nokia Bell Labs, 600 Mountain Ave, New Providence, NJ 07974 USA

**Keywords:** Micro-optics, Fibre optics and optical communications

## Abstract

Photonic lantern (PL) spatial multiplexers show great promise for a range of applications, such as future high-capacity mode division multiplexing (MDM) optical communication networks and free-space optical communication. They enable efficient conversion between multiple single-mode (SM) sources and a multimode (MM) waveguide of the same dimension. PL multiplexers operate by facilitating adiabatic transitions between the SM arrayed space and the single MM space. However, current fabrication methods are forcing the size of these devices to multi-millimeters, making integration with micro-scale photonic systems quite challenging. The advent of 3D micro and nano printing techniques enables the fabrication of freestanding photonic structures with a high refractive index contrast (photopolymer-air). In this work we present the design, fabrication, and characterization of a 6-mode mixing, 375 *µm* long PL that enables the conversion between six single-mode inputs and a single six-mode waveguide. The PL was designed using a genetic algorithm based inverse design approach and fabricated directly on a 7-core fiber using a commercial two-photon polymerization-based 3D printer and a photopolymer. Although the waveguides exhibit high index contrast, low insertion loss (−2.6 dB), polarization dependent (−0.2 dB) and mode dependent loss (−4.4 dB) were measured.

## Abstract (web summary)

A diminutive 3D nano-printed photonic lantern supporting multiplexing of six input sources to a six-mode optical fiber is demonstrated, at 375 μm total length, exhibiting low insertion, polarization and mode-dependent loss.

## Graphical abstract

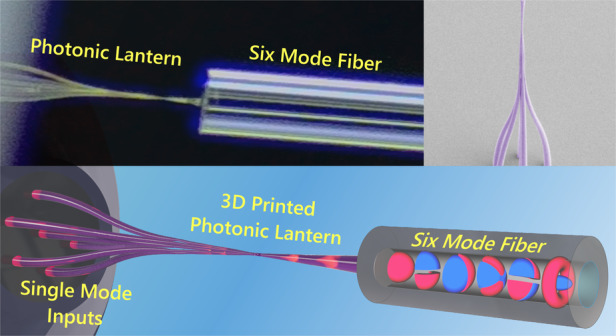

## Introduction

Spatial multiplexers find extensive applications in various light-related fields, including high-capacity mode division multiplexing (MDM) fiber communication networks^1–3^, free-space optical communication^4,5^, coherent power combining^6,7^, adaptive optics^8,9^ and wavefront sensing^10,11^. Spatial mode multiplexers (and demultiplexers) transform individual sources (modes) to spatially overlapping and orthogonal modes, ideally with low losses and in compact form. Photonic lantern (PL) mode multiplexers consist of an adiabatic (see definition in the “Discussion” section) spatial transition between a multi-mode optical waveguide and a discrete set of single-mode (SM) waveguides, with matching mode and waveguide counts^12^. They can losslessly convert from the multi-mode domain to the single-mode array domain and back and are one of the enabling technologies for MDM^13^. PL multiplexers can be categorized as either mode-preserving or mode-mixing devices. Both are compatible with fiber communication systems which require multiple-input, multiple-output (MIMO) equalization^14^ to unravel the mixing phenomena occurring in the fiber channel itself and possibly in the PL. PLs can be made by various means: SM fibers placed in a lower index sheath that coalesce to one^15–17^, by waveguide inscription in glass using direct laser writing^18,19^, and within photonic integrated circuits^20,21^. Due to the adiabatic transition requirement, PL devices are typically long and utilize low index contrast waveguides.

Three-dimensional (3D) nano printing techniques have revolutionized the field of photonics, enabling the fabrication of complex structures for manipulating optical waves with sub-micron resolution. This resolution is obtained by nonlinear two-photon polymerization (TPP)-based direct laser writing (DLW), for realizing optical structures with arbitrary shapes and sizes and excellent fidelity. Using TPP, 3D printed optical waveguides can be produced with high accuracy, allowing the creation of free-standing or nearly free-standing waveguides with lengths ranging from micrometers to millimeters. The technique creates air-cladded waveguides, which have low losses and can be used for many photonic applications^22–26^. Compared to DLW inside a glass medium, 3D nano-printing offers the advantage of fabrication on diverse materials and substrates, enabling versatile integration with different platforms. It provides the ability to achieve high coupling efficiency and good mode matching. Additionally, 3D nano-printing allows for the fabrication of high refractive index contrast waveguides (*n*_*polymer*_ = 1.53 vs. *n*_*air*_ = 1), with very small transverse dimensions required to remain single-mode^22^.

This work presents the design, fabrication, and characterization of a six-mode-mixing PL using 3D printing technology with a total length of only 375 *µm*. This diminutive PL includes waveguides for interfacing to six SM sources and an outgoing taper for interfacing and matching to a six-mode optical fiber. The spatial multiplexer (MUX) element within the PL, where the mode transformations occur, has a length of only 83 *µm*. The PL is a compact, free-standing device that we further support by an external structure designed to enhance its mechanical stability while minimizing any impact on performance. The PL’s unique design and fabrication technique allows for easy integration with a variety of sources and systems, as it can be 3D printed directly onto nearly any light source or other substrates, as in our case onto a multi-core fiber (MCF) end facet.

Table 1 provides a comparison of various mode multiplexers, encompassing all-fiber PL, glass-inscribed PL, and multi plane light conversion (MPLC) free-space technique. As depicted in the table, the 3D printed PL presented in this work exhibits mildly higher IL and MDL values compared to other multiplexers, which are attributed to fabrication imperfections. However, its size is roughly 10^2^ times smaller than the alternatives, offering a significant advantage in terms of direct integration onto diverse platforms and eliminating fiber interconnections that can affect footprint and size and possibly introduce differential fiber delays. Specifically, we envision the diminutive 3D printed PL seamlessly integrated with micro-scale integrated photonic circuits and dense VCSEL arrays. This integration feature enabled by additive 3D printing technology, along with its compactness and the ability to free-stand, makes it an ideal choice for diverse light-related applications extending beyond fiber-based systems.

**Table 1:** Comparison of different spatial mode multiplexer types

6-mode Multiplexer	IL	IL with FMF	MDL	Size	Source
All fiber PL	<0.7 dB	<0.7 dB	1.2 dB	125 mm long	^42,43^
Glass inscribed PL	<1.5 dB	<2.5 dB	–	50 × 15 × 10 mm	^35^
MPLC	>4 dB	–	1.2 dB	100 mm^3^ to 10 cm^3^	^44–46^
3D printed microscale PL	<3.3 dB, typical 2.67 dB	<5.4 dB, typical 4.8 dB	4.43 dB	Ø100 µm × H375 µm	This work

## Results

### Photonic Lantern design on a 3D nano-printing platform

The 3D printed PL device is made of a photopolymer that undergoes polymerization using TPP by a tightly focused writing laser beam of ultrashort optical pulses that scans the printing liquid volume point by point–or, more precisely, voxel by voxel–providing the ability to fabricate arbitrary three-dimensional few-micron-scale polymer waveguide structures with air cladding that are difficult—if not impossible—to fabricate using conventional techniques.

The SM sources in our demonstration originate from a MCF for the convenience of reduced source pitch of 35 *µm* and have a mode field diameter (MFD) of 6 *µm*, which are to be interfaced to a six-mode step index optical fiber with a core diameter of 15 *µm* and mode-dependent field diameters of 11.8–13.0 *µm*. The 3D printed waveguides exhibit a high refractive index contrast between core (*n*_*core*_ = 1.53) and cladding (air at *n*_*clad*_ = 1), therefore the six-mode waveguide size is 2.1 *µm* diameter (normalized frequency *V* = 4.69) and SM waveguide is 1 *µm* diameter (*V* = 2.35), at vacuum wavelength *λ*_0_ = 1.55 *µm*.

**Fig. 1:**
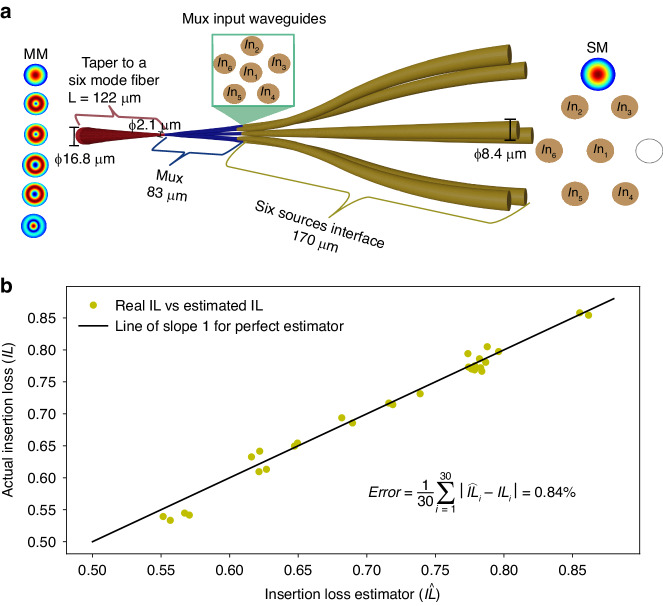
Photonic lantern layout. **a** The PL with a separation to its three components: Source interface, MUX and output taper. Right: Arrangement of the six input waveguides for the MUX. **b** IL computed from 12 FDTD simulations vs. IL estimator, computed with only 2 FDTD simulations

The PL contains three components (Fig. 1a): Waveguide interface section to match between the six-SM sources (pitch and size) to the multiplexer inputs, six-mode multiplexer (MUX), and an output taper to optimally match between the MUX output and a receiving six-mode optical fiber. Each element is separately designed and optimized. To create a 6-mode MUX that operates efficiently, the SM waveguides are configured in a pentagonal symmetry surrounding a central waveguide (Fig. 1a), which is the established arrangement for efficient excitation of six spatial modes^27^. Due to the high-index contrast waveguides, mode matching from the core sources to the mode size of the MUX’s inputs is required. Tapering down the waveguides from a diameter of Ø8.4 *µm*, which is optimized for coupling to the SM fiber core modes, to a SM polymer waveguide diameter (Ø1 *µm*) and then adiabatically expanding them to a 6-mode diameter (Ø2.1 *µm*) would necessitate long structures with very small diameter waveguides, jeopardizing the mechanical stability. In the present design, the source modes are tapered down to the input diameter of the multiplexer, denoted by an intermediate measure *R*. Subsequently, the waveguides continue to decrease in diameter within the MUX until reaching the final diameter supporting six modes at the output of the MUX. The main challenge in this design is avoiding the excitation of higher order modes. The mode matching taper, which is depicted as the “Six Source interface” in Fig. 1a, is optimized to maintain fundamental mode purity (with purity above 94%). Subsequently, the MUX is optimized based on the assumption that single-mode inputs are being introduced to its six inputs. We employed the Finite-Difference Time-Domain (FDTD) computation for the MUX simulation, which can be time-consuming. In order to calculate the insertion loss (IL) and modal dependent loss (MDL), a 12 × 12 coupling matrix must be computed to account for the six spatial modes and two polarizations, which involves conducting 12 FDTD simulations for each design iteration. It takes approximately 1 h to compute a single design’s coupling matrix using a computer with 16 CPU cores at 3.4 *GHz* and Lumerical FDTD solver. As a result, the optimization process can become nearly impossible to perform within reasonable time. Since the PL has azimuthal symmetry, we developed an IL estimator that allowed us to reduce the number of FDTD simulations required to two only (×6 speed up). For each iteration, we randomly choose a circumferential source and its X or Y polarized fundamental mode and the polarization of the central input source, and using these two input modes we find their respective PL output fields which are decomposed to the 6 × 2 eigen modes for computation of the coupling coefficients. Let *f*(*E*_1_) be the sum of the coupling coefficients when excited from the central core. Similarly, *f*(*E*_2_) is the coupling obtained by launching from a randomly selected circumferential core. In both cases, polarization state is chosen at random for each iteration. The IL estimator is:

1 $$\overline{{IL}}=\frac{2\cdot \left|f\left({E}_{1}\right)\right|+10\cdot \left|f\left({E}_{2}\right)\right|}{12}\le 1$$

The IL estimator operates under the assumption that the IL contribution from the central waveguide will differ from that of the circumferential waveguides. However, due to the symmetry of the circumferential waveguides around the center, their loss contribution is expected to be similar. As a result, the central waveguide |*f*(*E*_1_) | ^2^ is multiplied by a factor of 2 (accounting for 1 mode with 2 polarizations), while the result from the circumferential waveguides |*f*(*E*_2_) | ^2^ is multiplied by a factor of 10 (considering 5 modes with 2 polarizations). To test the estimator accuracy, we calculated the full coupling matrix for 30 different MUX designs and compared the actual IL to the estimation and found an average *L*_1_ error (see Fig. 1b) of only 0.84% between the estimator and the real IL (Fig. 1b). Our IL estimation method efficiently scales with the number of supported modes, drastically reducing the required simulation time. For example, when estimating the performance for a 15 mode-mixing PL, using an additional 9 sources arranged about an outer circle, only 3 FDTD simulations are required per iteration instead of 30 (compared to the 2 simulations required for the 6-mode case). If other design metrics such as crosstalk are required for optimization, the cost function can be expanded, as demonstrated in^28^.

#### Mux design

**Fig. 2:**
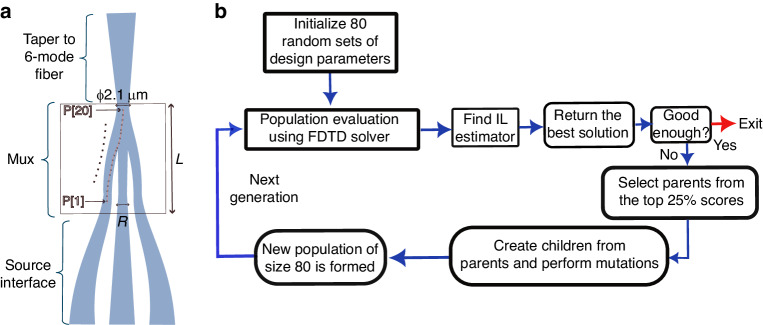
Photonic lantern design. **a** PL layout, with identification of the optimized design parameters: P,^1^P, ^2^…,P^20^ define the curve path, R is the input diameter of the PL (at termination of the source interface waveguides), and L is the MUX length. **b** Block diagram of the optimization process, using a genetic algorithm for efficient parameters update

3D fabrication technology enables writing complex structures^29^. To utilize the design space, we used a Genetic Algorithm (GA) implemented in Python and based on reference^30^ to optimize the MUX structure. The MUX input waveguides diameter (R), length (L), waveguides tapering form (exponential factor n) (Fig. 4c) and 20 points defining a curve were selected as the GA optimization parameters (Fig. 2a). We set the IL estimation function as an objective to be maximized. Then, the MUX was optimized by using the GA for efficient parameters update, along with FDTD simulations (Ansys Lumerical solver) for calculating each design objective function (Fig. 2b).

**Fig. 3:**
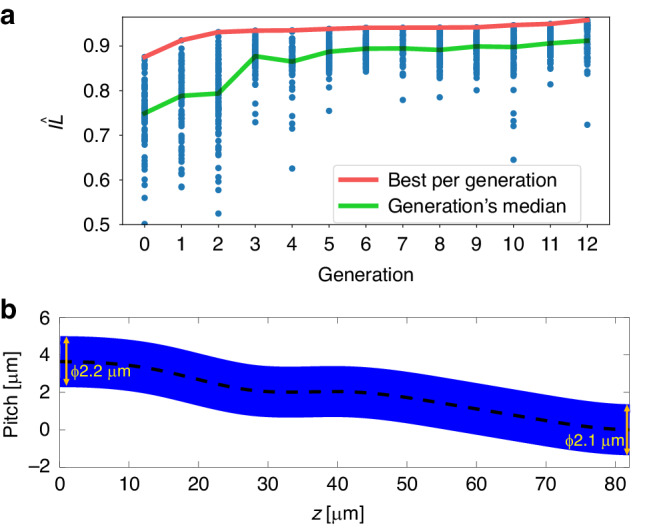
Photonic lantern design optimization **a** Genetic algorithm evolution throughout design generations. 80 variations (data points) shown per each generation and convergence for the best ${\widehat{{IL}}}$ score. **b** The optimized waveguides curve shape resulting from the GA optimization, with the I/O waveguide diameters

The GA design flow optimized the MUX, for an operation wavelength of 1.55 *µm*. Figure 3a illustrates the improvement of the IL across 12 generation cycles. In the plot, the blue points represent the 80 scores evaluated for each generation, the green line corresponds to the median score per generation and the red line tracks the best score achieved in each generation. The plot demonstrates consistent improvement in the best score measure for each generation cycle. The optimization process results in a design that achieves an ${\widehat{{IL}}}$ value of 0.96. Figure 3b displays the waveguide curve determined by the optimization process. The MUX has a length of 83 *µm* and an input diameter of Ø2.2 µm. Additionally, the pitch of the outer circle waveguides from the center was an optimization parameter, and it was set to 3.7 *µm*. The full 12 × 12 coupling matrix of the device was then calculated for comparison to the estimator. By calculating the singular values of the matrix (*λ*_*i*_) we can extract the IL and MDL by:

2 $${IL}=10\cdot {\log }_{10}\left(\frac{1}{N}\mathop{\sum }\limits_{i=1}^{N}{\left|{\lambda }_{i}\right|}^{2}\right)$$

3 $${MDL}=10\cdot {\log }_{10}\left(\frac{{\left|{\lambda }_{\min }\right|}^{2}}{{\left|{\lambda }_{\max }\right|}^{2}}\right)$$

We calculate an *IL* of −0.17 *dB* (96%) for the MUX, same as the ${\widehat{{IL}}}$, and *MDL* of −0.29 *dB*. The input cross-section diameter of the MUX is not single mode, hence excitation of higher order modes at the input facet may be an issue. In the simulation shown in the supplementary videos, the MUX was used for high-order mode de-multiplexing (operated in reverse), and the modes on the separate output waveguides are all single-mode, with different power distributions (and phases) between the waveguides.

#### Source interface waveguides design

**Fig. 4:**
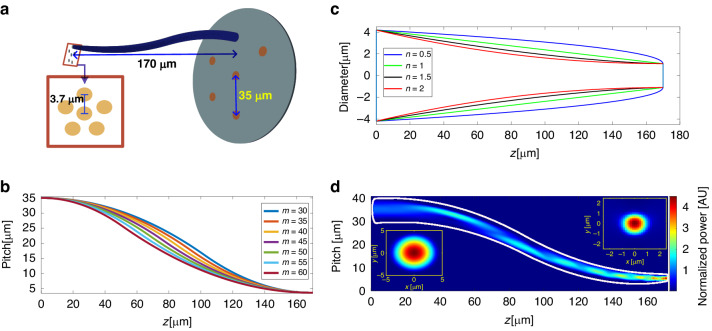
Source interfacing waveguides. **a** Waveguide transition from the 7-core fiber plane to the Mux input plane. **b** Visualization of the S-shaped curves with different m parameters. **c** Visualization of the taper’s shapes with different n parameters. **d** Side view of the pitch and diameter reducing waveguide, with its FDTD simulated light propagation and input/output mode distributions

In this work we used a 7-core fiber with a fanout device (Chiral Photonics^31^) as a source (using six of its cores). Each core is SM with MFD of 6 *µm* set at pitch of 35 *µm*. The designed diameter of the input waveguide for the MUX is 2.2 *µm*, which supports a fundamental mode with a MFD of approximately 1.8 *µm*. As a result, there is a large mismatch between the source mode with a diameter of 6 *µm* and the MUX input mode. Additionally, the pitch between the MUX input waveguides is 3.7 *µm* (Fig. 4a). To address this mismatch, we designed an S-shaped pitch transition taper to correct both the mode size and displacement. To achieve maximum coupling efficiency between the fiber core mode and the polymer waveguide fundamental mode (air-cladded), a waveguide diameter of 8.4 *µm* is required. The coupling efficiency is 97% in simulations. For taper optimization, we defined the waveguide tapering function as a power function of the form *x*^1*/n*^, where *n* is the parameter defining the tapering form (Fig. 4c). The second parameter, *m*, controls the S-shaped curve path where different values of *m* will generate a different curve as shown in Fig. 4b. The last parameter is the interface length. Longer slowly varying tapers should exhibit greater efficiency, but in practice they would be more difficult to fabricate, will introduce propagation loss due to surface scattering, and pose greater risk of not remaining intact after fabrication. We set a length of 170 *µm* for the source interface section as a compromise balancing performance and length.

By exploring the two-parameter optimization space, we found a solution (*n* = 0.7, *m* = 56) that achieved a mode purity of 95.2% for TE conversion and 94.8% for TM. The top right corner of Fig. 4d displays the mode shape at the taper output. After propagating through the waveguide, the mode shape remained that of the fundamental mode. Figure 4d shows the light propagation, the confinement of light in the waveguide indicates that the mode size reduction and bend radius change occurred gradually and smoothly with a minimal radius of curvature of 166 *µm*. Note that the central waveguide of the MUX is directly coupled to the central core of the fiber, requiring no displacement for this input waveguide, achieving a tapering efficiency of 99%. This leads to a power transmission imbalance between the central input waveguide compared to the outside waveguides having the S-bend.

#### Polymer waveguide taper to fiber LP modes

Since the polymer waveguide has high index contrast, its supported modes are not categorized as weakly-guided modes, as opposed to a fiber with low refractive index contrast. Matching the PL device output spatial modes to a 6-mode fiber requires optimizing the diameter of the polymer waveguide to best match a specific fiber. We selected a step index 6-mode fiber^32^ with a core diameter of 15 *µm* and a refractive index difference (∆*n* = *n*_*core*_ −*n*_*clad*_) of 9.7 × 10^−3^ for matching to our PL device. Since the PL supports dual polarizations and six spatial modes, it is crucial to optimize the cross-section diameter of the polymer waveguide for efficient coupling to the 2 × 6-modes. We calculated the coupling matrix between the polymer modes and the fiber modes for different waveguide diameters, and extracted the MDL and IL, targeting low MDL and high coupling efficiency.

**Fig. 5:**
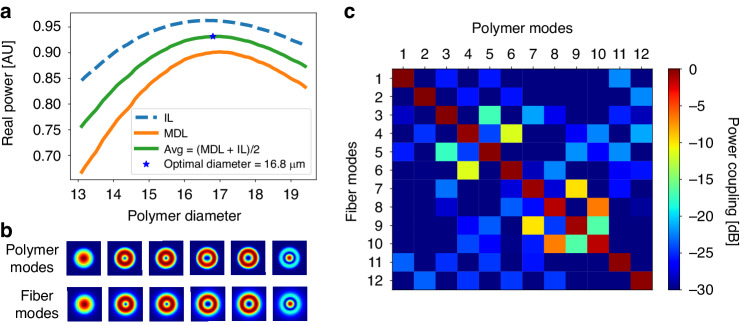
Mode matching between multimode polymer/air and glass fiber modes. **a** MDL and IL calculation for a range of polymer diameters. **b** Intensity profiles of polymer modes next to their corresponding LP fiber modes at optimal diameter. **c** Absolute squared coupling matrix between polymer and fiber modes

Figure 5a shows the MDL and IL for a range of polymer diameters. In this specific case, the MDL is calculated as $\frac{{\left|{\lambda }_{\min }\right|}^{2}}{{\left|{\lambda }_{\max }\right|}^{2}}$, and therefore, we are looking to maximize both metrics (IL and MDL). A diameter of 16.8 *µm* was chosen as the optimal diameter for the polymer waveguide to match the 15 *µm* core diameter optical glass fiber. This diameter achieved an IL of −0.2 *dB* and MDL of −0.5 *dB*. Figure 5b demonstrates a high similarity between the intensity profile of the fiber modes and polymer modes at the chosen diameter, leading to efficient coupling between them. The absolute squared coupling matrix to the selected diameter is almost diagonal, as shown in Fig. 5c, which further confirms the high similarity between the taper output modes and six-mode fiber.

**Fig. 6:**
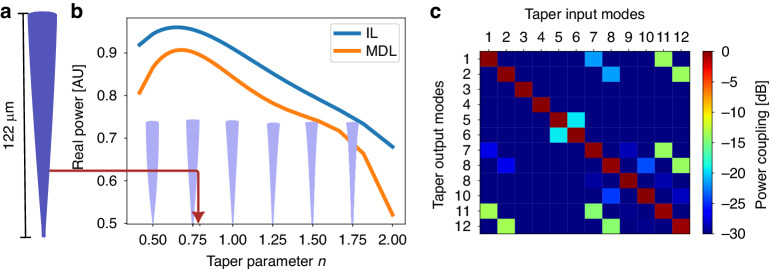
Multimode polymer taper. **a** The designed taper side view and dimensions. **b** MDL and IL calculation for a range of taper parameters. **c** Absolute squared coupling matrix between the taper input/output modes

To match between the PL and the fiber, we designed a taper which starts at the MUX output having a diameter of 2.1 *µm* (polymer’s 6-modes waveguide) and ends with a diameter of 16.8 *µm* having a V number of 39.4 supporting $N\approx \frac{{V}^{2}}{2}=777$ modes. We limit the taper length to 115 *µm*, with the first 7 *µm* of the taper set at constant 2.1 *µm* diameter to serve as filter for higher order modes^33^ that may be excited by the multiplexer. The transmission of higher order modes through the 7 *µm* long mode filter is lower than −20 *dB*. The adiabatic taper design should prevent the excitation of higher-order modes while keeping its length relatively short. The optimization parameter in this case is the taper form, which is defined as a power function and shown in Fig. 4c. Figure 6b shows the MDL and IL as a function of the tapering form. The plot shows an optimal performance point at *n* = 0.6 (*IL* = −0.2 dB and *MDL* = −0.4 dB). However, this taper is more massive and top-heavier, making it susceptible to stress at its base and deformation. We back off slightly and chose the value of *n* = 0.8 (Fig. 6a), which still gives excellent performance (IL of −0.3 dB (94%) and an MDL of −0.5 dB (89%)), yet reduces the taper mass by 5%.

#### Complete device performance

**Fig. 7:**
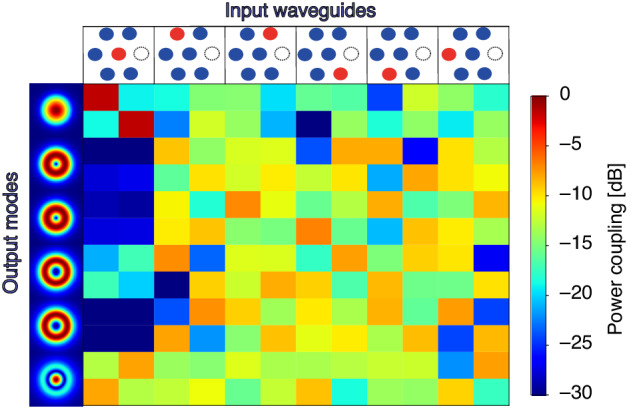
Photonic lantern’s power coupling matrix. Simulation results of input-output modes' coupling values (magnitude squared, in dB scale)

To evaluate the performance of the complete PL mode multiplexer, we simulated the full 375 *µm* long device, comprising the source interface, the MUX, and the output taper, and calculated its coupling matrix elements. From this matrix, an IL of −0.8 *dB* and MDL of −1.4 *dB* were found. Figure 7 shows the coupling matrix absolute value squared, where the terms *In*_*i*_^*x/y*^ are referring to the different SM inputs (Fig. 1b) with two orthogonal polarizations (*x/y*). Since the multiplexer is designed as a mode scrambling device, it is expected that the power will be spread across the modes, and therefore a diagonal matrix is not *x*/*y* expected. The power coupling matrix shows highest efficiency for inputs *In*_1_ due to the fact that this particular waveguide does not undergo any bends throughout the entire device, resulting in minimal loss and higher coupling efficiency predominantly to the fundamental mode.

### Fabrication of photonic lantern

**Fig. 8:**
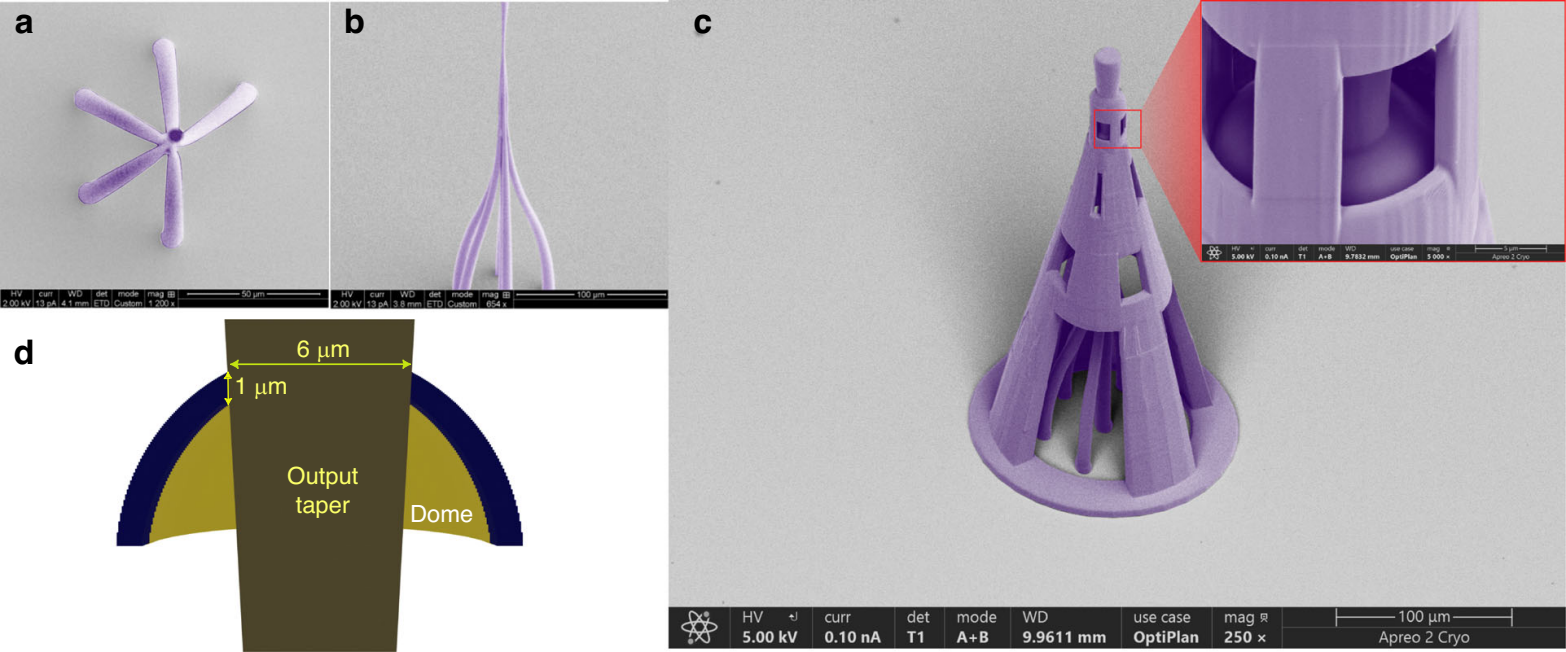
Fabricated photonic lanterns. **a**, **b** SEM top and side views of prototype PL structure on glass substrate. **c** SEM image of PL structure with a supporting frame. **d** Sliced side view of the supporting dome area at output taper, from 3D CAD model

A prototype PL was initially fabricated on a glass substrate (Fig. 8a, b). For a detailed description of the process, see the “Methods and Materials” section. Since the PL has a relatively long length and narrow diameter waveguides, its structural integrity cannot be guaranteed. Hence a support structure was designed to prevent undesired bends and deformations of the structure (Fig. 8c). To minimize any impact on the optical performance, the support structure is interfaced with the PL via a 1 *µm* thin polymer layer structured as a hollow dome (Fig. 8d). The interface point is at a waveguide taper diameter of 6 *µm*, where the six intended modes are tightly confined within the waveguide, and any interaction between the dome shell and the guided mode is negligible. The dome contributes a loss smaller than 0.01 *dB* in our simulations.

### Characterization of the PL

#### Off axis digital holography

**Fig. 9:**
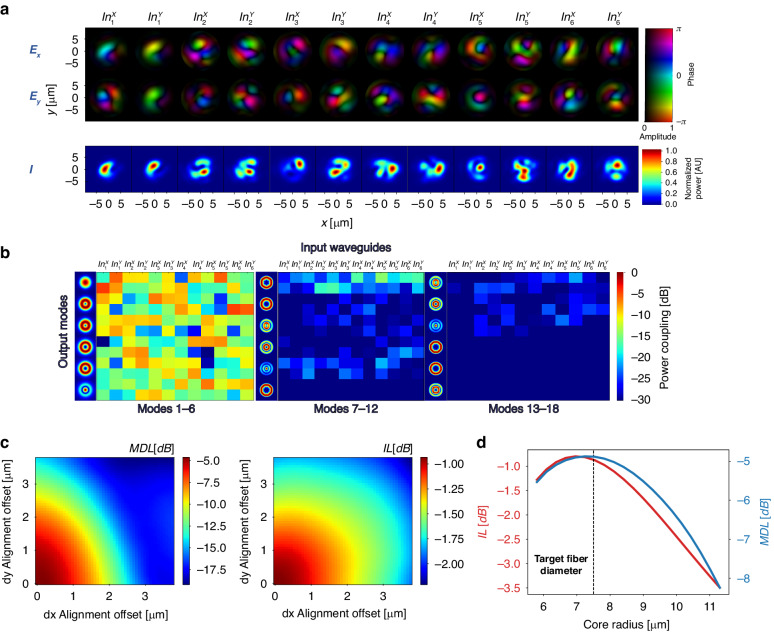
Photonic lantern output field characterizations. **a** The reconstructed electric fields resulting from each input mode of the PL contain two orthogonal complex field components (E_x_ and E_y_). **b** The absolute squared values of the coupling matrix are shown with respect to the 6-mode fiber modes (left), waveguide modes 7–12 (middle) and waveguide modes 13–18 (right). **c** IL and MDL as a function of fiber offset in x and y directions. **d** IL and MDL as a function of target fiber core radius

Off-axis digital holography was employed (refer to the Methods and Materials section) to measure the complex electric field at the output of the PL for each input mode excitation and determine the coupling matrix by performing mode decomposition with the 6 target fiber modes. This approach emulates fiber coupling without encountering technical challenges such as cleaving, butt coupling, and other associated alignment complexities. After calculating the coupling matrix, we find the MDL and IL by utilizing singular value decomposition (SVD), as in Eqs. (2) and (3). Figure 9a shows all of the reconstructed electric fields used for calculating the coupling matrix. For each excitation, the complex *E*_*x*_ and *E*_*y*_ are shown, along with the intensity $I={|Ex|}^{2}+{|Ey|}^{2}$.

From the measured coupling matrix, whose magnitude squared values are shown in Fig. 9b, an IL of −0.9 *dB* and MDL of −4.4 *dB* were calculated. A notable disparity exists between the simulated MDL value of −1.4 *dB* and the measured MDL. This mismatch is likely attributable to fabrication errors. The supplementary materials contain an analysis of two potential types of variations that can occur during the fabrication process, from which we see that MDL is more sensitive to the examined variations. Since the PL taper output supports higher order modes $\left(V=39.4\to N\approx \frac{{V}^{2}}{2}=777\right)$, we check whether higher order modes are excited with mode decomposition using an additional 12 spatial modes (24 with polarization). As shown in Fig. 9a, b, the highest coupling value to one of these higher order modes is −15 *dB*, indicating their low excitation.

The PL should be aligned to a matched MM fiber at its end, hence we evaluate how potential misalignment and mode mismatch will affect the MDL and IL metrics. This is done using the output field analysis of the fabricated device, rather than simulated fields. By shifting the centroids of the virtual fiber modes relative to the PL, we gain insight into the effects of misalignment.

Similarly, by changing the virtual fiber core diameter and mode basis, we assess the impact of size mismatch. Figure 9c illustrates how the MDL and IL change as a result of misalignment in both the x and y directions. The data shows that the MDL is more sensitive to misalignment than the IL, with a degradation of over 10 dB observed across the range of [0 − 4]*µm* offset values tested. Figure 9d shows the MDL and IL as a function of fiber core diameter. The optimal point, in terms of MDL, is achieved at a fiber core diameter of 15.2 *µm*, while the optimal core diameter is 14.4 *µm* for IL. Recall that a fiber with core diameter of 15 *µm* was employed as a target fiber for the PL design.

#### Output power and transmission through a 6-mode fiber

**Fig. 10:**
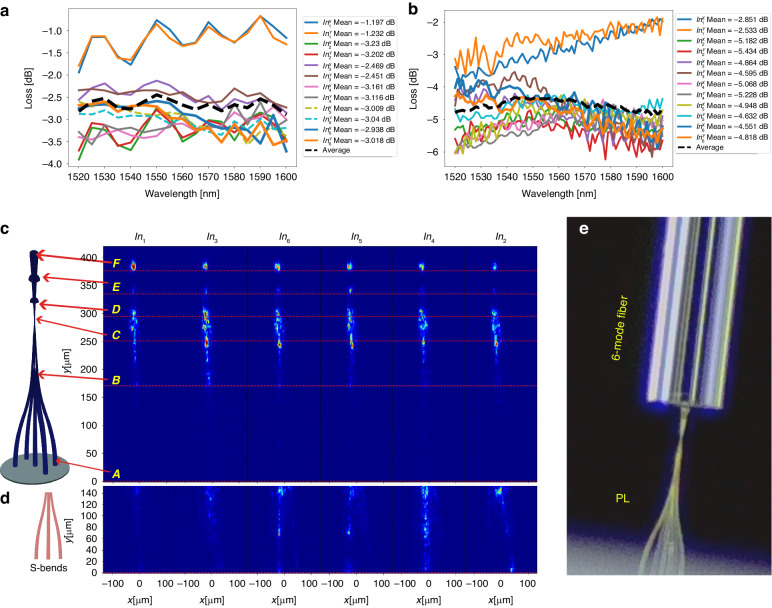
Photonic lantern losses to free-space and to fiber. **a** Output power loss vs wavelength per input mode. **b** Output power following butt-coupling to a 6-mode fiber. **c** IR images of the PL from a top view for identifying scattering, with each input excited and significant points on the PL marked on the images. **d** IR images of the section "F"-"A" with higher exposure time. **e** Microscope image of the PL butt coupled to the fiber

Projecting the PL’s output onto a 1*cm*^2^ free-space InGaAs power meter, the PL’s output optical power transmission per input source/polarization excitation is evaluated. Figure 10a depicts all 12 measurements of the PL optical power loss, following calibration to account for the losses associated with the optical components in the setup. A wavelength range of [1520–1600] nm has been tested. The setup is described in “Methods and materials” (Fig. 14b top). The output optical power difference between polarization states for each input is negligible, with a maximum polarization dependence of less than 0.2 dB. The loss per input is even throughout the selected wavelength band. The graph also displays the average loss curve, with a mean value of −2.7 dB. The discrepancy between the most efficient curve and the least efficient curve is about 2 dB, where the central waveguide input corresponds to the most efficient curve, as there are no bends in this waveguide path throughout the entire structure. In simulations under ideal conditions, the efficiency difference between the central waveguide input and the outer ones is 0.6 dB on average (computed by a summation of each column elements in the simulated coupling matrix in Fig. 7). The difference between the most efficient waveguide (*In*_1_) and the second most efficient one (*In*_3_) is 1.2 dB. To investigate the reason behind the discrepancy in input efficiency, we utilized an IR camera attached to a microscope to obtain side-view images of the PL (Fig. 14c). We visualize the side-scattered light from each input in the image. The regions with high side scattering correspond to lossy regions, as a perfectly guided wave would be invisible in this case.

Figure 10c illustrates the IR camera images captured for each of the PL inputs, sorted from left to right based on their efficiency measurements. A properly scaled 3D CAD image of the PL is displayed alongside. The white horizontal lines, red arrows, and letters A-F indicate different regions of the PL structure in the IR images. The “F” line corresponds to the output of the PL, where we expect most of the light. The images captured by the IR camera (Fig. 10c) show a strong correlation between the spots above the F-line and the power measurements. The “C” line marks the MUX output. In this section and in the 7 *µm* long mode filter, the waveguide supports 6 modes only. It is noteworthy that the scattering light in this region for *In*_1_ image is much smaller than other inputs, indicating a greater excitation of higher order modes by the circumferential waveguides compared to the central one. This difference in excitation directly translates to power loss. The positions of the thin support domes that connect the PL are labeled as “D” and “E”. Examining point “E” in the images reveals scattering from the domes, where the effect is pronounced for *In*_5_, *In*_3_ and *In*_6_. One explanation for the large scattering in the dome area for these specific inputs can be attributed to the output field resultants from these inputs. According to the holography analysis, the coupling matrix (Fig. 9b) shows a relatively large coupling coefficient to the highest order mode. Therefore, the field diameter will be larger in this case and the interaction of the evanescent wave with the dome is more likely to occur.

It appears that there is minimal scattering effect between points “A” and “B,” which correspond to the “Source interface waveguides”. To determine if there are any differences between the inputs in this specific region, we observed it with longer exposure time as shown in Fig. 10d. The lower amount of scattered light observed in the images for inputs *In*_1_ and *In*_3_ compared to the other inputs helps account for the power measurement differences shown in Fig. 10a. This observation suggests that the power degradation in this case may be attributed to fabrication imperfections rather than a design issue. Analyzing the “A” line, which represents the interface plane between the PL and the 7-core fiber source, provides insight into the coupling between the two components. Misalignment or mode mismatch can result in coupling issues. Examining Fig. 10d, the image of *In*_2_ reveals significantly higher scattering at the interface plane compared to the other inputs. This likely indicates a misalignment between the fiber core and the 3D printed polymer waveguide. This observation can also provide an explanation for the high losses measured from this input.

Output power measurements assess transmission efficiency, however for a spatial mode multiplexer the coupling efficiency to a 6-mode fiber is the most relevant metric. In an additional experiment, butt-coupling was performed (Fig. 10e) between the PL and a specialized, higher refractive index contrast, 1 *m* long step-index 6-mode fiber with a core diameter of 10 *µm*. For this subsequent experiment, the taper was shortened and ended at a fiber-matched diameter of 12.4 um. Coupled optical power was measured at the fiber’s distal end (Fig. 14b bottom), for all input modes and evaluated wavelengths (Fig. 10b). In comparison to Fig. 10a, all measurements exhibited a reduction ranging from 1.7 to 2.2 *dB*, with the wavelength-averaged loss per mode decreasing from −2.7 *dB* to −4.8 *dB*. From the off-axis digital holography measurement, an IL of −0.9 *dB* was determined. Given that the digital holography emulates the scenario of perfect fiber coupling, the additional losses likely result from coupling imperfections as finite gap, tilt, alignment, etc., and are estimated to be approximately −1.4 *dB* when calculated based on the average curves in both plots.

#### Optical Vector Network Analyzer (OVNA) measurement

**Fig. 11:**
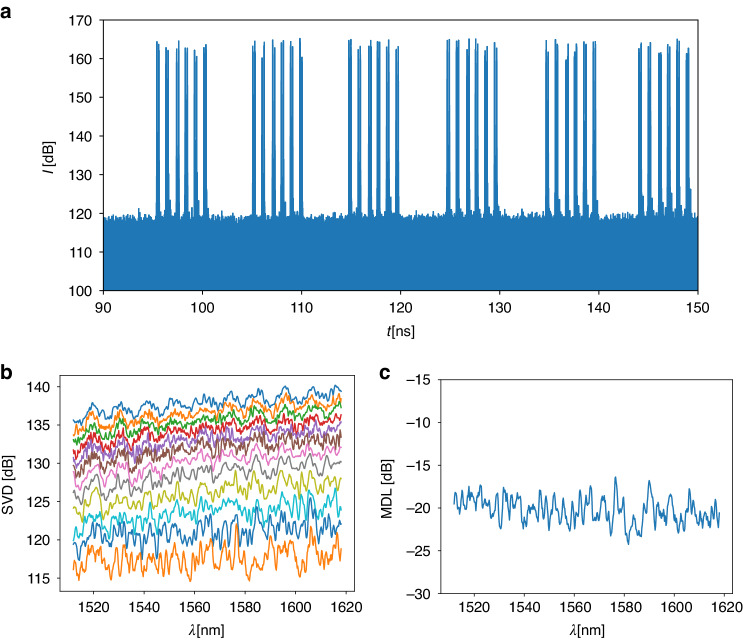
System experiments, with the PL MUX, 6 mode-fiber, and a mode-preserving DEMUX. **a** OVNA measurement intensity in the time domain. **b** 12 SVD values as a function of wavelength. **c** MDL as a function of wavelength, calculated from the values in (**b**)

System transmission tests using the micro-scale PL as a MUX, a six-mode fiber segment, and a fiber-based mode-preserving PL as a DEMUX^34^ (Fig. 14d), were performed with an optical vector network analyzer (OVNA) measurement of the system transfer matrix (additional information in “Methods and material”). The intensity of the OVNA measurement in the time domain is presented in Fig. 11a. Using encoded time delays by different path length fibers, the impulse responses *h*_*i,j*_ are clearly distinguishable from each other, with a total of 2 × 36 peaks observed in the measurement, indicating that all 2 × 6 modes were effectively excited by our 6-mode mixing PL. After applying time windowing on the measurement, we generated the time-domain impulse response *h*(*t*) of the system, as shown in the supplementary materials. We then extracted the frequency response, *H*(*ω*), using the FFT.

The singular values of *H*(*ω*), per wavelength, are presented in Fig. 11b, which show stability across the measured spectrum. MDL was calculated from the SVD (difference of max and min values), at −20 dB level (Fig. 11c). While the utilized 6-mode preserving PL combined with the 6-mode fiber have been reported to contribute an MDL of approximately 1.2 *dB*^35^, we believe the main reason for the low MDL is due to the fiber alignment and size mismatch between the microscale PL and the fiber. As shown in Fig. 9c, the MDL is highly sensitive to misalignment, and even a potential tilt between the fiber and the PL can have an impact. We remind that the 3D printed PL was designed for a 6-mode fiber with a 15 *µm* core diameter, whereas in the system setup the core diameter of the fiber was 18 *µm*, resulting in a mode mismatch contribution.

## Discussion

**Fig. 12:**
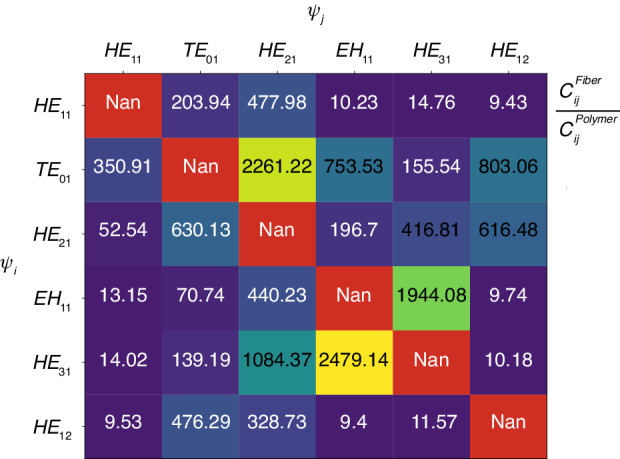
Study of adiabatic condition fulfillment. The ratio between the adiabatic criteria for fiber modes and polymer modes, for all 36 mode combinations

PL devices and other mode multiplexers tend to be bulky and cumbersome. This is primarily due to the utilization of weakly guided modes in these multiplexing devices, which necessitates the use of long devices to maintain an adiabatic transition. A “strictly adiabatic” system is characterized by the maintenance of its original eigenmode. In the case of a PL, a more relaxed criterion states that the energy within the system eigenmodes should predominantly stay within those modes. In this relaxed case, the system can remain lossless, with power shifting between its eigenmodes^12^. Let us examine the adiabatic condition within a taper, given by^36^:

4 $$\left|\frac{2\pi }{{\beta }_{i}-{\beta }_{j}}\frac{d\rho }{{dz}}\right.\left.\int {\psi }_{i}\frac{\partial {\psi }_{j}}{\partial \rho }{dA}\right|\ll 1$$

where *ψ*_*i*_ and *ψ*_*j*_ are the normalized field distributions of system modes *i* and *j*, ${\beta }_{i}$ and ${\beta }_{j}$ are their respective propagation constants, *ρ* is the varying parameter, in our case the core diameter, and *z* is the longitudinal coordinate along the PL or taper. The tapering rate is therefore $\frac{d\rho }{{dz}}$. As apparent in Eq. (4), it becomes more challenging to satisfy the adiabatic condition when the propagation constants of the modes are close to one other. The utilization of high index contrast and small diameter waveguides, which increase the spacing between the propagation constants of guided modes^36^, enables the miniaturization of the PL device from millimeters to micrometers. To better understand the differences in adiabatic criteria between a weakly guiding waveguide and relatively high contrast polymer waveguide as in this work, we compare a 6-mode fiber as described in^32^ with a 6-mode polymer waveguide having a diameter of 2.1 *µm*. The design process and the utilization of multiple degrees of freedom provided by 3D printing technology allow us to control the tapering function *ρ*(*z*), thereby controlling the term $\frac{d\rho }{{dz}}$ in Eq. (4). Our focus is on the term and $\frac{2\pi }{{\beta }_{i}-{\beta }_{j}}$, also known as the beating length^37^. Due to a larger spacing in the propagation constants of the polymer compared to the fiber, we anticipate a significant advantage for the polymer in terms of beat length. However, the polymer waveguide modes vary more compared to those of the fiber, which implies that the integral term might be larger (change of mode per change of core radius). To evaluate this, we performed a numerical calculation based on the analytical solution of circular waveguides^37^ for the relevant quantity ${C}_{{ij}}=\left|\frac{2\pi }{{\beta }_{i}-{\beta }_{j}}\int {\psi }_{i}\frac{\partial {\psi }_{j}}{\partial \rho }{dA}\right|$. For both the polymer and fiber cases, we calculated *C*_*ij*_ for all possible combinations of the 6 modes, resulting in a total of 36 calculations. Let ${C}_{{ij}}^{{polymer}}$ and ${C}_{{ij}}^{{fiber}}$ denote the results for the polymer and fiber, respectively, with modes *i* and *j*. Each element in the matrix depicted in Fig. 12 represents the ratio $\frac{{C}_{{ij}}^{{polymer}}}{{C}_{{ij}}^{{fiber}}}$ for a specific mode combination. Since all values are greater than 1, it indicates that the adiabatic criteria for the large index contrast polymer waveguide are easier to satisfy compared to those of the fiber. Overall, we observe a reduction in the range of 10^1^ to 10^3^.

The implementation of 3D printing technology in the design and fabrication of free-standing multi-micron PL devices has the potential to revolutionize the utilization of spatial multiplexing in novel applications and platforms. The capability to print on diverse substrates and materials facilitates compact integration with micron-scale components like VCSEL arrays, silicon photonic integrated circuits, and other photonic technologies. This advancement enables enhanced flexibility and opens up new avenues for compact, integrated photonic systems with improved functionality and performance.

In conclusion, we designed, fabricated and characterized a six-mode photonic lantern with a total length of 375 *µm* using high refractive index, DLW waveguides, while the MUX alone is 83 *µm* long only. The device showed power losses of −2.7 *dB*, indicating the efficiency of power transfer in the PL, and IL of −0.9 *dB* and MDL of −4.4 *dB* when calculating its coupling to a matched virtual multimode fiber. Due to its short length, wavelength-dependent effects experience minimal evolution, resulting in nearly uniform performance of the device across the wavelength range of 1510 − 1620 *nm*. Future work will focus on refining the fabrication process, improving fiber alignment techniques, and increasing the number of modes to 10 and beyond.

## Materials and methods

### Simulation tools

The electromagnetic simulations carried out using Ansys Lumerical solvers encompass the utilization of FDTD, EME, and FDE algorithms. The optimization and design process involves utilizing a Python API that has been developed in-house for the Lumerical solver.

### 3D printing process

A NanoScribe Photonic Professional GT printer was utilized in the fabrication process, employing IP-Dip photoresist, which is specifically formulated to match the refractive index of the microscope objective that focuses the laser beam. This results in an ideal laser beam focusing and fine lateral resolution (200 nm) of the fabricated structures^38^. Prior to fabrication, a salinization process was used to enhance polymer adhesion to the optical fiber facet (silica). The fabricated structure was developed in PGMEA (Propylene glycol monomethyl ether acetate) for 20 min and then cleaned with IPA (Isopropyl Alcohol) for 2 min, and drying with Novec 7100 for 1 min. To achieve the desired shape and size of the waveguide with minimum surface roughness, we optimized various writing parameters, such as laser power set to 35% of the maximum (Nanoscribe’s laser), scanning speed of 10,000 µm/s, and the distance of laser scanning hatching set to 0.05 µm (lateral spacing between lines) and slicing set to 0.1 µm (spacing between layers in the z-axis). The fabrication time for the PL structure is approximately 20 min, while an additional 2 h is required for the support structure.

### Printing on a 7-core fiber

In this work, a 7-core fiber having a pitch of 35 *µm* and a single core MFD of 6 *µm* was utilized. The fiber, supplied by Chiral Photonics, was equipped with a fiber fanout to 7 single-mode fibers. In order to print the micro-scale PL onto the tip of a 7-core fiber with each input aligned to a single core, an alignment procedure must be carried out prior to printing.

**Fig. 13:**
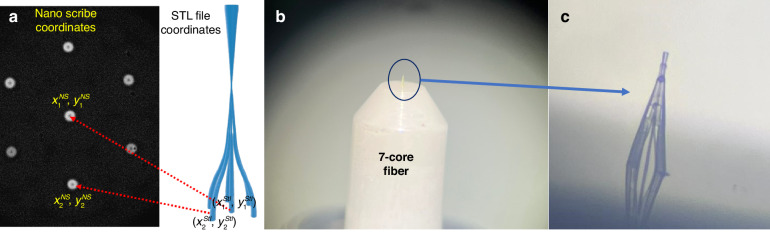
Printing on a MCF tip. **a** Left − 3D CAD of the PL structure with its coordinates notation; Right - Microscope image of the fiber cores with the coordinates notation. **b** Microscope image of the printed PL on top of the 7-core fiber. **c** Magnified image of the printed structure on the fiber core

The NanoScribe printer coordinates (*x*^*NS*^*,y*^*NS*^) and the PL’s STL file coordinates (*x*^*STL*^*,y*^*STL*^) are denoted, as shown in Fig. 13a. As the outer cores of the fiber exhibit hexagonal symmetry, a simple approach is to select two points from the STL file and map them to their corresponding points in the Nanoscribe coordinate space for accurate alignment. If there is a deviation in the core position of the outer cores from the central core, it can lead to misalignment. Following measurements, we found the maximum core positional error to be 0.5 *µm*. Such misalignment, given the 6 *µm* MFD, results in excess loss of only −0.12 *dB* in power coupling efficiency for the fundamental mode.

### Off-axis digital holography

**Fig. 14:**
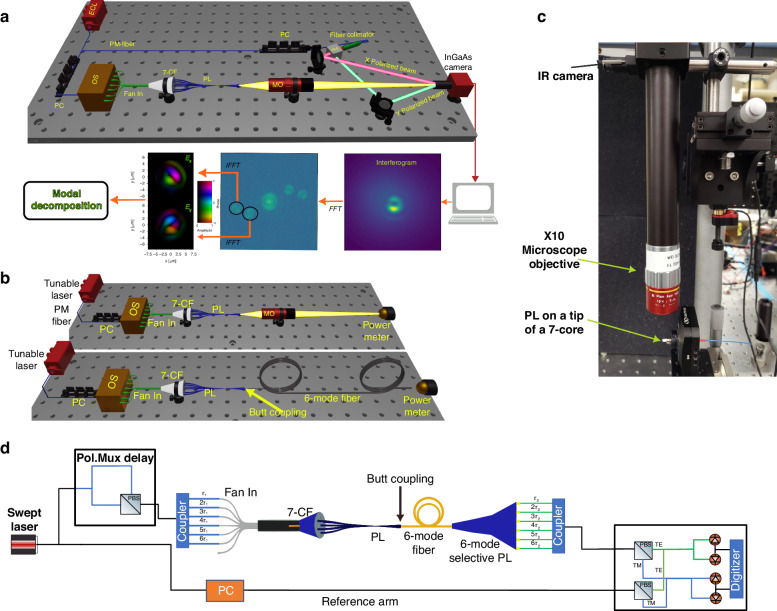
Experimental arrangements for device characterization. **a** Off-axis holography set-up. PC Polarization Controller, PL Photonic Lantern, OS optical switch, PM Polarization Maintaining, 7-CF 7-core fiber, M: ×20 Microscope Objective with a tube lens of *f* = 200 *mm*; PBS Polarization Beam Splitter, FFT Fast Fourier Transform. **b** Top: direct power measurement set-up; Bottom: Transmission measurement through a 6-mode fiber. **c** IR imaging set-up for analyzing loss sources in the structure. **d** OVNA set-up

To measure the coupling matrix of the PL for a single wavelength (1.55 *µm*), we employed off-axis digital holography to capture the complex electric field of the device’s output. Using two orthogonally polarized reference beams (X and Y polarized) enables capturing the two interference field components simultaneously, denoted as *E*_*x*_ and *E*_*y*_ respectively. Using a polarization controller before the PL, we launch two orthogonal input modes to each of the PL inputs depicted as *In*^*x*^_*i*_ and *In*^*y*^_*i*_ where *i* ∈^1–6^. We then performed modal decomposition (MD) using 12 digital modes that were simulated and supported by the 6-mode fiber (with polarization). The optical setup is shown in Fig. 14a and the digital signal processing (DSP) process is depicted. More details about the method above can be found in ref. ^39^. In our experimental setup, we utilized the following equipment: a Yenista optics model −1560 ECL (External Cavity Laser) source, Thorlabs MPC320 polarization controllers, Thorlabs TC25FC-1550 fiber collimator for collimating the reference beam, a ×20 T1.1 Mitutoyo LCD Plan Apo NIR (Near-Infrared) infinity corrected objective with a 200 *mm* tube lens for focusing the output beam of the PL onto the camera plane, and an Allied Vision Goldeye G-033 TECless InGaAs camera.

### Direct power measurement

Figure 14b illustrates the configuration used to measure the power transmission efficiency. The setup included a Yenista optics model −1560 tunable ECL source, which operated across the wavelength range of 1520–1600 *nm*, and an HP-8153A optical power meter. To account for losses caused by optical devices such as the optical switch and fanout, we initially measured the power transmission of the entire system using the 7-core fiber, excluding the PL structure. This measurement was performed on six cores, with each core having two polarization states, resulting in a total of 12 baseline measurements for the system. Each measurement vector covered the required wavelength range. We denote the measurement of core *i* with input polarization *x*\*y* as $P{S}_{i}^{{x\backslash y}}$.

After fabricating the PL on the measured fiber, we repeated the same measurement procedure, measuring the power emerging from the PL device. We denote these measurements as ${{PL}}_{i}^{{x\backslash y}}$. The loss measurement of the PL per input *i* and polarization, $x\backslash y$ (as shown in Fig. 10a), was calculated using the normalization formula and converted to dB scale:

5 $$P{L}_{i}^{x/y}=10\cdot {\log }_{10}\left(\frac{{PP}{L}_{i}^{x{{\backslash }}y}}{P{S}_{i}^{x{{\backslash }}y}}\right)\left[{dB}\right]$$

### Optical vector network analyzer (OVNA)

Swept wavelength interferometry is a technique that can be used to measure the polarization resolved complex transfer function *H*(*ω*) and impulse response *h*(*t*) of an optical device under test^40^. To minimize the effect of fiber coupling, we used a 6-mode selective PL^41^ spliced to a 6-mode fiber as a demultiplexer device. Therefore, it was necessary to butt couple the micro-scale PL to the 6-mode fiber that is attached to the demux device. The setup for OVNA measurement is shown in Fig. 14d, where the micro-scale PL is directly aligned to a short 6-mode fiber which is further spliced to the 6-mode selective PL. We set the delay constants *τ*_1_ and *τ*_2_ to 1 *ns* and 10 *ns*, respectively. Swept laser range is 1510–1620 *nm*. The alignment of the micro-scale PL to the 6-mode fiber was performed using a microscope with ×10 magnification and 2-xyz stages.

### Supplementary information

Supplementary materials

Excitation of mode 1 from MM end

Excitation of mode 2 from MM end

Excitation of mode 4 from MM end

Excitation of mode 5 from MM end

Excitation of mode 7 from MM end

Excitation of mode 9 from MM end

Excitation of mode 10 from MM end

Excitation of mode 12 from MM end
