# Supplementary material for: Free-standing microscale photonic lantern spatial mode (De-)multiplexer fabricated using 3D nanoprinting
Source: Light Sci Appl. 2024 Jun 3;13:126. doi: 10.1038/s41377-024-01466-6 (PMC11144700; doi:10.1038/s41377-024-01466-6)
Supplement: Supplementary file 1 — Supplementary materials [file 41377_2024_1466_MOESM1_ESM.pdf]

Supplementary materials for "Free-Standing Microscale Photonic Lantern Spatial Mode (De-)Multiplexer Fabricated using 3D Nanoprinting"

Yoav Dana[1,*], Yehudit Garcia[1], Aleksei Kukin[1], Lauren Dallachiesa[2], Sterenn Guerrier[2], Nicolas K Fontaine[2], and Dan M. Marom[1]

[1]Institute of Applied Physics, Hebrew University of Jerusalem, Jerusalem, Israel
[2]Nokia Bell Labs, 600 Mountain Ave, New Providence, NJ 07974, United States of America
[*]yoav.dana@mail.huji.ac.il

Fabrication errors analysis

To evaluate the device's performance under more realistic conditions involving fabrication errors, we conducted additional simulations. Various scenarios were simulated, and the resulting MDL and IL were computed for each case. These simulations offer a more accurate assessment of the device's performance in practical applications and can guide the development of fabrication strategies to minimize performance degradation. During the 3D printing process, an increase in the diameter of the waveguides is likely to occur due to the overlap of VOXELs. Another crucial consideration is the potential for structural shrinkage in the longitudinal axis during the post-printing development process. To explore the effects of these phenomena, we simulated the device under various cases involving shrinkage and increments in waveguide diameter. For each case, we calculated the coupling matrix and extracted the MDL and IL. Figure.1 presents a plot of the MDL and IL against the two types of variations tested. The selected range for the shrinkage factor is $0-10\%$, and for the waveguide diameter increment, it is $0-17\%$. Under the examined effects, the MDL has the potential to decrease by 3.4 dB, and the IL by 1.5 dB. It's important to note that numerous additional factors could contribute to performance degradation, including surface roughness and waveguide deformation due to mechanical stress. Nevertheless, predicting and simulating such effects is inherently more complex.

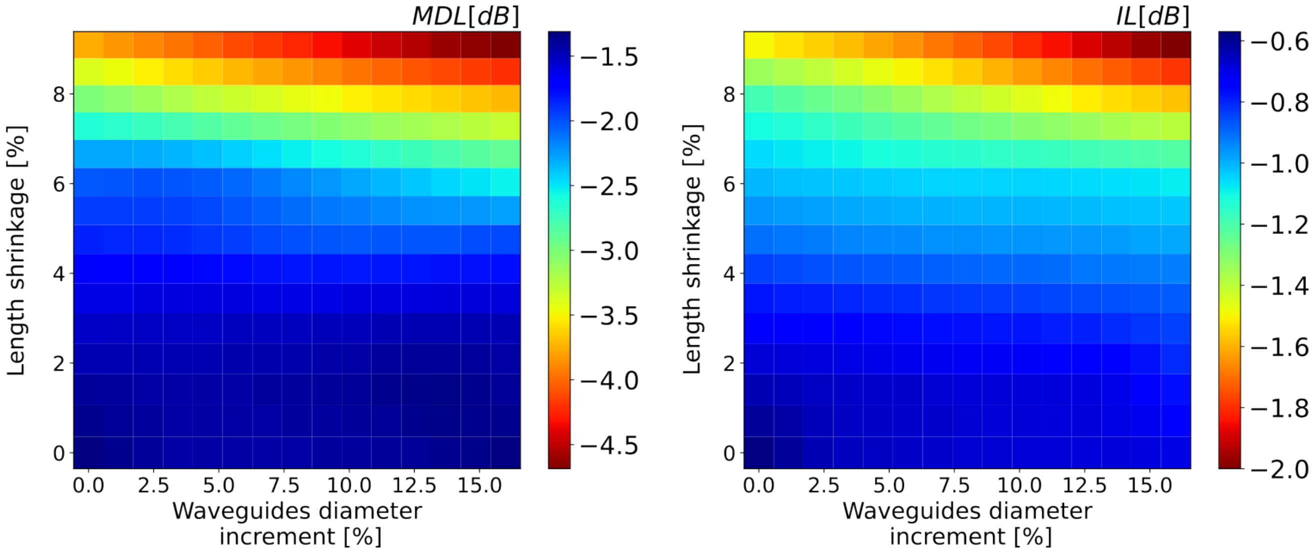

Figure 1. left-MDL [dB],right-IL [dB] vs shrinkage percentage and waveguides increment percentage.

Impulse response OVNA measurment

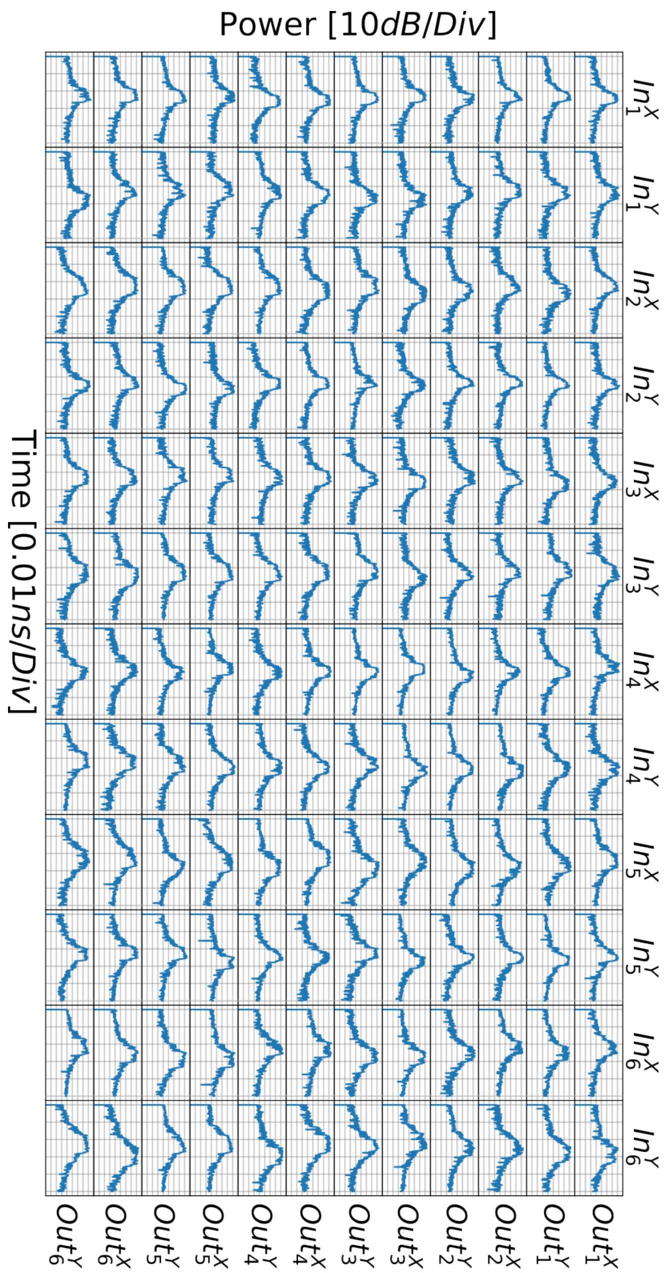

Figure 2. Time response impulse of the six-input, six-output system, consisting of our microscale PL as mode mux, segment of 6-mode fiber, and a fiber-based PL as mode demux. The columns represent the microscale PL input modes, and the rows represent the mode-selective PL modes used as the Demux.

Modal decomposition of output fields

In Figure 3, the power coupling coefficients for each output field, measured using the off-axis digital holography method with an operating wavelength of 1.55 μm, are illustrated. We have selected the modes of a 6-mode fiber with a core diameter of 15 μm as the modal basis for digital demultiplexing. Additionally, each plot title includes the sum of all coefficients. Values exceeding 0.79 signify that the field predominantly comprises the six targeted output modes.

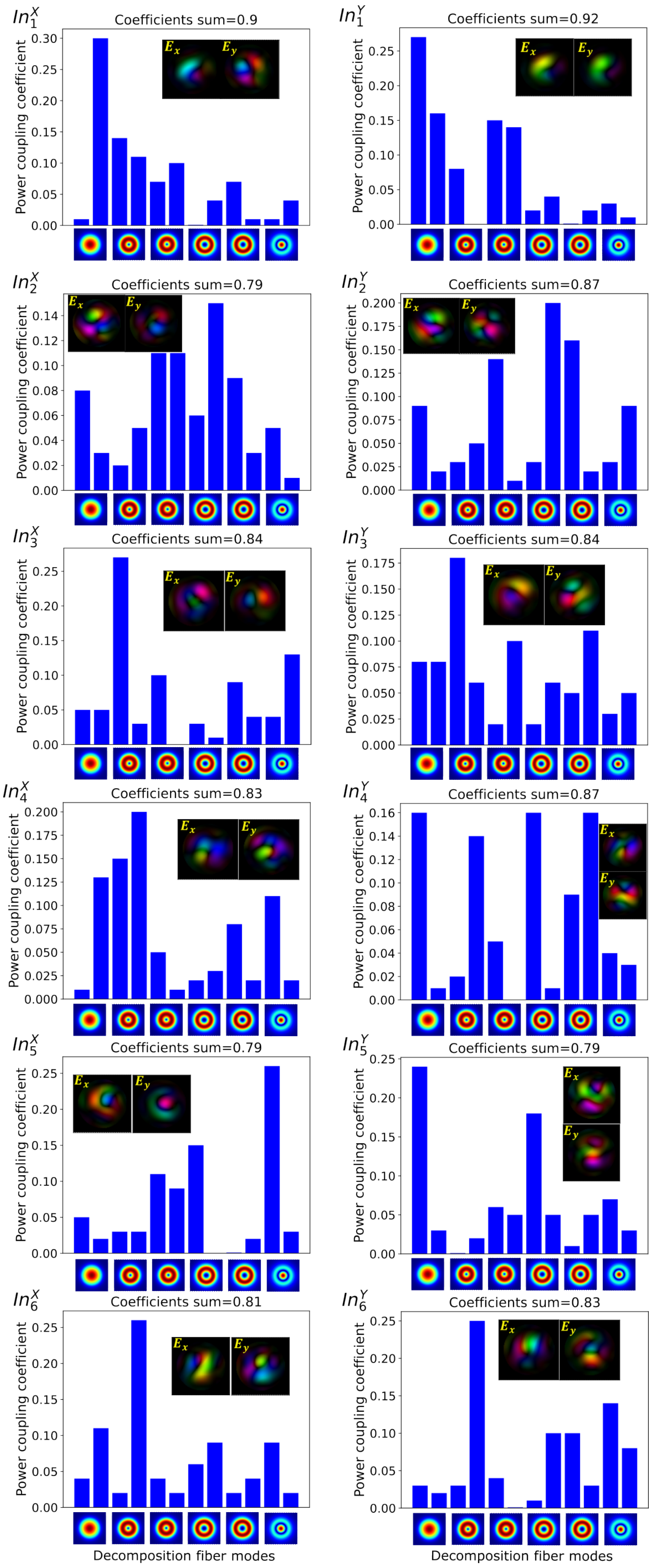

Figure 3. Modal decomposition of each complex measured output field of the PL.
